# Uric Acid, Colchicine and Chronic Inflammatory Diseases: A Cardiovascular Perspective

**DOI:** 10.3390/metabo15070424

**Published:** 2025-06-20

**Authors:** Alessia Alunno, Francesco Carubbi, Claudio Ferri

**Affiliations:** Department of Clinical Medicine, Life, Health and Environmental Sciences, Internal Medicine and Nephrology Division, ASL1 Avezzano-Sulmona-L’Aquila, San Salvatore Hospital, University of L’Aquila, 67100 L’Aquila, Italy

**Keywords:** uric acid, colchicine, gout, rheumatoid arthritis, psoriatic arthritis

## Abstract

Based on the notion that inflammation plays a pivotal role in the development and progression of cardiovascular diseases (CV) and that hyperuricaemia is an independent CV risk factor, chronic inflammatory diseases such as gout and rheumatoid arthritis are an interesting case study. Both conditions are burdened by an excess CV risk; they are themselves an independent CV risk factor, and in the case of gout, hyperuricaemia is a hallmark of the disease. Colchicine, a drug historically used for the management of gout, has recently been repurposed for secondary CV prevention in individuals at high CV risk. The purpose of this review article is to discuss evidence on CV diseases and CV prevention in rheumatoid arthritis, gout, and other chronic inflammatory/systemic autoimmune diseases with a focus on inflammation and hyperuricaemia.

## 1. Introduction

Uric acid (UA) is a molecule produced through the metabolic breakdown of endogenous and exogenous purine nucleotides and this process is catalysed by the enzyme xanthine oxidoreductase. UA is a waste product, and as such, it is eliminated by the kidneys (two thirds) and by the small intestine (one third) [1].

Hyperuricaemia is defined as serum UA (SUA) levels above 6.0 mg/dL in women and above 7.0 mg/dL in men and it can be either asymptomatic or associated with a variety of clinical features including arthritis (gout), tophi or nephrolithiasis [2]. A number of different conditions, drugs and dietary habits may cause hyperuricaemia, accounting for excessive UA production, decreased UA excretion or both. However, an unhealthy diet, with or without impaired renal UA excretion, is the most frequent cause of hyperuricaemia.

It is now well established that the clinical relevance of SUA levels goes well beyond the simple association with gout and/or nephrolithiasis [3]. Several studies pointed to hyperuricaemia as a CV risk factor in the general population; therefore, the 2018 guidelines of the European Society of Cardiology (ESC) and the European Society of Hypertension (ESH) included SUA assessment among the screening tests to be performed in hypertensive patients [4]. Furthermore, in view of the association between hyperuricaemia, mortality (both CV and all-cause) and CV events, it is conceivable that cardiovascular damage begins with SUA levels lower than 6 mg/dL.

Besides SUA, another leading actor in the development and progression of CV diseases in the general population is inflammation [5]. In patients with chronic inflammatory diseases such as gout, rheumatoid arthritis (RA) and psoriatic arthritis (PsA), the inflammatory burden is even more pronounced than in the general population, accounting for an excess CV risk in these individuals [6,7]. Although gout, RA and PsA have a completely different pathogenesis, the underlying mechanisms of systemic inflammation overlap to some extent. In addition, ongoing subclinical inflammation and synovitis, as observed in RA and PsA, have also been demonstrated in asymptomatic patients with gout, reinforcing that in gout, the acute inflammatory response of disease flares builds on an underlying chronic inflammation [8].

The purpose of this review article is to discuss the relationship between hyperuricaemia, inflammation and CV risk/events in gout, the paradigm inflammatory disease caused by persistently elevated SUA levels, and other chronic inflammatory diseases.

## 2. Uric Acid and Cardiovascular Risk: An Overview

The association between uric acid and CV diseases has been increasingly investigated over the last 60 years, revealing a link between elevated SUA levels and a variety of conditions including, among others, hypertension, metabolic syndrome, cerebrovascular disease and vascular dementia [9]. For example, elevated SUA levels are observed in up to 60% of patients with untreated arterial hypertension and in up to 90% of adolescents with essential arterial hypertension of new onset. These findings, alongside evidence from studies comparing patients with hypertension of different origin (e.g., white-coat vs. primary vs. secondary), point to UA as a key factor in the development of early primary hypertension [10]. Of interest, although SUA levels are often associated with metabolic syndrome, hyperuricaemia is not included among the diagnostic criteria that have been proposed internationally for the definition of this pathology and whether it should be included in the future is still a matter of debate. A meta-analysis published in 2015 showed that for every 1 mg/dl increase in SUA level, the risk of metabolic syndrome increases by 30% with a linear dose–response relationship [11]. A simple reason for the association between SUA and metabolic syndrome is the mere result of the association between SUA and the single components of metabolic syndrome. However, as this is a multifactorial disease, the linearity of the association between SUA levels and the risk of metabolic syndrome likely underscores other aspects yet to be clarified.

Since hyperuricaemia often coexists with (and even triggers/worsens) other CV risk factors, the identification of its net and direct contribution to CV risk may be a clinical challenge. Nonetheless, the Uric Acid Right for Heart Health (URRAH) study identified an SUA threshold value of 4.7 mg/dL for all-cause mortality and 5.6 mg/dL for CV mortality [12]. In addition, in the same study, SUA was an independent risk factor for cerebrovascular events after adjusting for potential confounding variables, including arterial hypertension, and identified a valid prognostic cut-off value (>4.79 mg/dL). Concordantly, SUA levels >5.34 mg/dL (sensitivity 52.32 and specificity 63.96) were the univariate prognostic cut-off value for all heart failure, whereas SUA levels > 4.89 mg/dL (sensitivity 68.29 and specificity 49.11) were the cut-off value for fatal heart failure [13]. This relationship was still significant in multimorbid patients with metabolic syndrome, reinforcing the role of SUA in the CV setting.

On this basis, urate-lowering therapies are an interesting facet of CV prevention strategies. A recent meta-analysis including 18,585 hyperuricaemic patients from 30 randomised controlled trials (RCTs) showed that SUA reduction with xanthine oxidase inhibitor therapy resulted in a reduced incidence of major adverse cardiovascular events (MACE) [14]. In more detail, by including twelve trials on febuxostat (8261 patients) and seven trials on allopurinol (1217 patients), the authors observed that each 1 mg/dl reduction in the SUA level was associated with an average 6.0% CV risk reduction. However, a subsequent meta-analysis specifically focused on allopurinol and also including the results of large randomised controlled trials (RCTs) such as the ALL-HEART [15], shed additional light on the matter [16]. The authors showed that despite the fact that allopurinol did not improve a number of CV outcomes in the overall cohorts, the benefit in patients with acute coronary syndromes or undergoing coronary artery bypass grafting compared to placebo was evident.

Regarding CV death, the pooled analysis of ten studies (6665 patients) reported no significant reduction of CV mortality (risk ratio (RR) 0.60; 95% confidence interval (CI) 0.33–1.11) in patients treated with allopurinol vs. placebo in addition to the standard of care. When focusing on the five studies enrolling patients undergoing coronary artery bypass grafting, a significant reduction of CV death was observed but only in the short term after the procedure (≤30 days, RR 0.27; 95% CI 0.10–0.77). This result was no longer significant in the medium and long term.

Concerning all-cause mortality, although a substantially reduced rate was observed among the patients undergoing coronary artery bypass grafting (RR 0.31; 95% CI 0.12–0.79), no significant difference emerged in the overall cohorts.

Finally, when focusing on CV events, despite an impressive short-term (≤30 days) benefit of allopurinol vs. placebo in preventing post-procedural myocardial infarction in patients undergoing coronary artery bypass grafting (RR 0.29; 95% CI 0.09–0.94), no significant effect was observed in the overall patient cohorts. A lack of benefit in the overall cohort was also reported for MACE and for stroke.

It is worth mentioning that the authors also compared allopurinol to febuxostat and no differences emerged since the RRs for CV outcomes were similar. In this regard, the Febuxostat for Cerebral and Cardiorenovascular Events Prevention Study (FREED) trial compared febuxostat with non-febuxostat with a composite primary outcome including CV events, mortality and renal function. Although febuxostat did not affect CV events, the decline in renal function was inhibited in those allocated to febuxostat as compared with usual therapy. To note, the non-febuxostat arm used 100 mg allopurinol/day, complicating the interpretation of the results [17].

Data on CV outcomes of uricosuric drugs are scarce and often conflicting [18].

Despite evidence on the CV role of elevated SUA levels and on the CV impact of SUA lowering, at least in some patient subgroups, to date there is no recommendation supporting the pharmacological treatment of asymptomatic hyperuricaemia for CV prevention purposes.

For the sake of completeness, it is worth mentioning that other drugs that are not included in the family of urate-lowering therapies have shown an effect on SUA levels. For example, meta-analyses of randomized trials have also shown that sodium/glucose cotransporter 2 (SGLT-2) inhibitors used in patients with diabetes reduce SUA levels in various clinical scenarios [19,20,21]. Although the exact mechanisms of the SUA-lowering effects of SGLT-2 inhibitors are not entirely clear, these agents may reduce UA levels via the inhibition of UA production and/or by increasing UA excretion [22].

## 3. Inflammation and Cardiovascular Risk: An Overview

In normal conditions, the endothelium has anti-inflammatory and antithrombotic properties, and regulates the permeability to circulating molecules and the vascular tone through the balance between the release of vasodilator substances, such as nitric oxide, and endothelium-derived constrictors. In the presence of triggers that reduce the bioavailability of nitric oxide (e.g., traditional CV risk factors and uric acid), these protective properties are lost and endothelial permeability is increased. This leads to a subendothelial accumulation of cholesterol-containing lipoproteins which ultimately induces a low-grade inflammatory response. The endothelial inflammatory response encompasses the activation of both innate immunity (macrophages) and adaptive immunity (T- and B-lymphocytes and dendritic cells) [23,24]. The peculiar role of inflammation in the CV scenario has prompted the evaluation of anti-inflammatory agents in addition to conventional treatment for the management of CV risk factors for secondary prevention. Canakinumab, a fully human monoclonal antibody against IL-1β, was found to be able to significantly reduce MACE and mortality [25], whereas methotrexate failed to show any benefit on CV risk [26]. Both drugs were burdened by adverse events such as fatal infections (canakinumab), increased liver enzymes and reduced leukocytes (methotrexate). Furthermore, canakinumab also posed economic issues due to the cost of sustained therapy over time. At present, other IL pathways are under investigation in CV prevention, for example, RCTs exploring the IL-6 antagonist ziltivekimab are ongoing [27,28].

In this scenario, colchicine proved to be a cost-effective therapeutic option with an acceptable safety profile. The results of the Low-Dose Colchicine (LoDoCo) and LoDoCo-2 trials [29,30], showing that by adding colchicine to the standard of care in patients with stable coronary disease, MACE and CV death were reduced in the long term, led to the approval of colchicine at the dosage of 0.5 mg once daily in patients with established atherosclerotic disease or with multiple risk factors for CV diseases [31]. Based on these trials, and even before the formal approval by the Food and Drug Administration, colchicine was already mentioned in international guidelines as a possible additional treatment in patients with either sub-optimal control of other risk factors or in those with recurrent CV events despite optimal CV risk management [32].

## 4. Uric Acid and Gout

The natural history of gout involves the progression from hyperuricaemia to the deposition of monosodium urate (MSU) crystals [33]. Elevated SUA levels are a necessary, but not sufficient, condition for MSU crystal deposition, and it is still unclear why this deposition happens only in some individuals with hyperuricaemia. In people with gout, an acute inflammatory response to deposited MSU crystals typically presents as a self- limiting arthritis that affects the joints of the lower limb (gout flare). MSU crystals are preferentially deposited at the first metatarsophalangeal joint, the midfoot and, to a lesser extent, the knee. The gout flare involves increased production of pro-inflammatory cytokines, mainly IL-1β, by macrophages and monocytes, and the infiltration of neutrophils into affected tissues. Gout flares usually resolve spontaneously within 7–10 days. If untreated, chronic inflammation and tissue remodelling lead to the development of tophi, inflammatory granulomatous tissue with deposited MSU crystals and ultimately to irreversible joint damage (chronic gout) [34].

The prevalence of gout ranges between 2.7 and 6.7% in countries with a western lifestyle and it is progressively increasing overtime. The so-called “westernization” of lifestyle, characterised not only by incorrect eating habits but also by an increase in sedentary life, facilitates the development of hyperuricaemia and, possibly, gout, but also of several metabolic (e.g., obesity, type 2 diabetes mellitus and/or dyslipidaemia) and CV comorbidities. Since patients with gout are burdened by a higher CV risk compared to the general population, based on the above-mentioned evidence, it is plausible that by reducing SUA levels to prevent gout flares, an effect on the CV side should also be expected. Allopurinol is broadly used to reduce SUA levels in chronic gout and with the approval of febuxostat in the late 2000s, another weapon was added to the armamentarium for the management of chronic gout [34]. However, evidence on the CV effects of xanthine oxidase inhibitors in patients with gout has been conflicting and often derived by observational studies enrolling mixed populations with and without gout [35].

Over the last decade, two pivotal RCTs in this area have been published, the Cardiovascular Safety of Febuxostat and Allopurinol in Patients with Gout and Cardiovascular Morbidities trial (CARES) [36] and the Long-term Cardiovascular Safety of Febuxostat Compared with Allopurinol in Patients with Gout Trial (FAST) [37]. Both trials aimed to compare allopurinol and febuxostat with CV safety as a primary outcome in patients with gout; however, the CARES study was a double-blind RCT whereas the FAST study was a non-inferiority open-label trial. In the CARES study, although allopurinol achieved a less profound reduction of SUA levels as compared with febuxostat, patients had lower CV and all-cause mortality. Conversely, in the FAST study, no significant differences in any CV outcomes were observed in patients treated with allopurinol or febuxostat. To note, all patients included in the CARES trial had a history of previous CV disease as compared with only thirty-three percent of patients in the FAST trial. The early release of safety data from the CARES trial led the Food and Drug Administration to issue a warning first in 2017 and then in 2019 limiting the use of febuxostat only to patients not tolerating or not responding to allopurinol.

In addition, data from large real-life cohorts published in the same period were in line with the results of the CARES trial rather than with those of the FAST trial, underlining the importance of the careful use of febuxostat in selected patients but also proving that the CARES recruitment strategy was a true reflection of real-life settings [38,39].

## 5. Uric Acid and Rheumatoid Arthritis

Historically, the occurrence of gout in patients with rheumatoid arthritis (RA) has been rarely investigated, mainly due to the notion that RA and gout were mutually exclusive conditions. However, over the last decade, the number of studies exploring this area has increased, shedding some light not only on the burden of gout but also of asymptomatic hyperuricaemia in RA.

In fact, the demonstration of the role of SUA as a CV risk factor in the general population and the increasing knowledge on excess CV risk in RA fuelled the interest in investigating SUA in this disease as well. A recent study using the large National Health and Nutrition Examination Survey (NHANES 1997–2018) database found that overall, patients with RA had higher SUA levels compared with individuals without RA and the prevalence of hyperuricaemia in RA was 27.4% compared with 18.2% among the general population [40]. The authors identified an association between hyperuricaemia and RA but subgroup analysis results showed inconsistent associations across different subgroups. In more detail, the association was significant in the subgroups of individuals of female sex, aged 60 years or above, non-Hispanics, those with hypertension, those using antihypertensive drugs and those with a body mass index (BMI) of 30 kg/m^2^ or above. Of interest, disease duration, but not age at diagnosis, significantly influenced this relationship. In fact, the association between RA and hyperuricaemia was significant in patients with a disease duration between 15 and 30 years but not in those with a shorter or longer disease duration.

The observation that up to one third of RA patients may show hyperuricaemia, regardless of coexistent gout, reinforces the relevance of previous studies demonstrating a strong association between SUA levels and CV diseases in RA.

In fact, in 2008, Panoulas et al. showed that RA patients with CV events had higher SUA levels compared to patients without CV events [41]. Of interest, they also showed an association between SUA levels that remained significant even after adjustment for traditional CV risk factors such as hypertension, smoking pack-years, total cholesterol, obesity and insulin resistance indices, as well as other potential confounders, such as renal function, the use of drugs and RA disease severity, suggesting that SUA may be independently associated with CVD [odds ratio (OR) = 1.36, 95% confidence interval (CI) 1.04–1.79, *p* = 0.025] [42].

More recently, a strong association between SUA levels and peripheral arterial events has been reported. This association was again still significant after adjusting for demographic data (age and sex), CV risk factors (smoking, hypertension, obesity, diabetes mellitus and dyslipidaemia) and other confounders (renal function and urate-lowering therapy) [hazard ratio (HR) = 2.54; 95% CI = (1.13–5.70)]. In addition, this study revealed a trend towards a stronger association with mortality among RA patients [HR = 1.96; 95% CI = 1.45–2.65) than among individuals without RA (HR = 1.57; 95% CI = 1.09–2.24) [43].

With regard to overt gout, a recent study showed that it could be detected in 17% of RA patients [44]. Of interest, this study also detected an association between hyperuricaemia and CV mortality, but this was no longer significant when adjusting for comorbidities and for disease-specific variables such as disease activity.

Another interesting facet of hyperuricaemia in RA pertains to the possible involvement of specific pro-inflammatory mediators and to the pathogenic events occurring at the lung level. In 2011, a population-based study enrolling over 6000 individuals demonstrated that SUA levels were directly correlated with IL-6 and tumour necrosis factor (TNF)-α circulating levels, and therefore, SUA levels were linked to the extent of chronic inflammation [45].

Furthermore, studies conducted in animal models of lung inflammation showed that UA can be released to the bloodstream from injured lung cells [46]. In the same period of time, pivotal studies from various groups put forward the hypothesis that the pathogenic immune reactions leading to RA development may be initiated at sites other than the joints, and that the lungs could harbour such sites [47]. Lastly, a recent systematic review showed that patients with interstitial lung disease (ILD) associated with autoimmune diseases, including RA, had a 1.65 times increased risk of CV events compared with patients without pulmonary manifestations of the autoimmune disease [48].

Taken together, these findings suggest that lung inflammation has a role in determining CV risk in patients with RA, and since inflamed lung cells may produce UA, contributing to hyperuricaemia, a link between hyperuricaemia, lung inflammation and CV risk in RA is an intriguing new scenario worth further investigation.

## 6. Uric Acid and Psoriatic Disease

The term psoriatic disease encompasses a clinical continuum of cutaneous, musculoskeletal and other systemic manifestations, including CV diseases. The evolution from psoriasis (PsO) to psoriatic arthritis (PsA), usually defined in studies by the point at which the patient meets the Classification Criteria for Psoriatic Arthritis (CASPAR), is a multistep process involving several genetic and environmental factors [49]. It is now well-established that patients with psoriatic disease display a higher CV risk compared to the general population, but unlike RA, the metabolic rather than the inflammatory component seems to be predominant in determining this increased risk [7].

The association between hyperuricaemia, gout and PsO has been extensively investigated over time, highlighting a prevalence of hyperuricaemia of up to 50% of patients with PsO and up to 30% in patients with PsA. A meta-analysis showed that patients with PsO had a 2.56-fold higher risk of hyperuricaemia [odds ratio (OR) = 2.56, 95% CI (1.82–3.59)], whereas patients with PsA had a 3.56-fold higher risk of hyperuricaemia [OR = 3.56, 95% CI (2.04–6.20)] [50].

Of interest, in psoriatic disease, additional sources of UA have been described as contributing to such a high prevalence of hyperuricaemia in this disease (even higher in patients with cutaneous manifestations). Increased epidermal cell proliferation and turnover, as observed in PsO, accelerates purine nucleotide catabolism thereby leading to increased UA production [51]. In addition, patients with psoriatic disease often display metabolic-dysfunction-associated steatotic liver disease (MASLD) (formerly non-alcoholic fatty liver disease, NAFLD) and studies conducted in patients with NAFLD have revealed an increased production of UA from the hepatocytes [52].

Despite this high prevalence of hyperuricaemia in psoriatic disease, the studies assessing the coexistence of psoriatic arthritis and overt gout, the so-called ‘psout’, are scarce and mainly individual case reports [53].

Only a few studies have explored the link between hyperuricaemia and CV risk in psoriatic disease, and this may be due to the difficulty in quantifying the role of individual metabolic CV risk factors that coexist in the majority of patients [7]. In this regard, Gonzalez-Gay et al. showed that in patients with PsA without CV risk factors, there was a significant association between high SUA and subclinical atherosclerosis [54], whereas AlJohani et al. found an increased prevalence of CV disease, mainly myocardial infarction and congestive heart failure, in PsA patients with hyperuricaemia [55].

Owing to the complexity of the metabolic scenario in PsA and the mutual relationship between individual conditions, the real burden of hyperuricaemia and the benefit of reducing SUA for CV prevention in psoriatic disease remains unclear.

## 7. Uric Acid and Other Chronic Inflammatory Diseases

Data regarding hyperuricaemia and gout in other autoimmune inflammatory conditions such as systemic lupus erythematosus (SLE), systemic sclerosis (SSc) and Sjogren disease (SD) are very few. Gout is rarely observed in SLE, with a reported prevalence < 5% [56], and data on hyperuricaemia in SLE showed an association of higher SUA levels with various clinical features as well as with overall damage accrual but specific data on the CV area are lacking [57,58,59].

Likewise, elevated SUA levels in SSc have been associated with disease-related vascular damage but evidence on CV risk is lacking [60].

Conversely, we recently demonstrated a strong relationship between SUA levels, CV risk and disease manifestations in SD [61]. In more detail, when classifying SD patients according to SUA levels using the cut-off values of the URRAH study, we identified specific subgroups characterized by a different metabolic profile (worse in those with higher SUA levels) but also by different disease features. We observed a relationship between SD-ILD and CV events and in multivariable analysis, this relationship was dependent on SUA levels ≥4.79 mg/dL but independent of other traditional CV risk factors such as hypercholesterolemia and obesity. Our data fit with the above-mentioned notion that lung inflammation may be a key player in UA homeostasis and ultimately in CV risk [46]; however, these findings require confirmation in larger longitudinal studies.

## 8. Inflammation and the Cardiovascular Repurposing of Colchicine in Gout and Other Chronic Inflammatory Diseases

Gout is a chronic inflammatory disease and as such, a suggested element in the context of the relationship between gout and CV disease is IL-1 [33,34]. Although multiple soluble mediators of inflammation have been implicated in the pathogenesis of the acute episode of MSU crystal arthritis, IL-1β is the key molecule behind this phenomenon. The inflammasome is a multi-protein cytoplasmic complex consisting of various elements including a ‘sensor’ protein, adaptor proteins and proteins with an enzymatic function, the caspases. The presence of stimuli that interact with the sensor protein triggers a cascade of events culminating in the activation of caspase-1 and the production of IL-1 β from its precursor (pro-IL-1β). Although MSU crystals can activate the inflammasome and therefore induce IL-1β release, the notion that not all hyperuricaemic individuals develop acute gouty arthritis despite MSU crystal precipitation, has led to the understanding that the full activation of the inflammasome requires a second hit.

This additional stimulus may be represented by a load of free fatty acids (which would explain why a large meal is a risk factor for an acute gouty episode), or by microbial-derived products such as lipopolysaccharides [33]. The activation of the inflammasome also leads to the release of another key molecule, IL-18. However, IL-18 does not appear to be directly involved in the genesis of the joint inflammatory process in gout as it is in other inflammasome-mediated diseases, such as adult Still’s disease, where IL-18 is as involved as IL-1 in determining the clinical manifestations [62]. However, both IL-1β and IL-18 play an important role in the context of CV risk and therefore the activation of the inflammasome in gout opens up new scenarios worthy of discussion.

To date, in fact, there is solid evidence that IL-1 contributes both to the evolutionary development of atherosclerotic plaques and their ischemic complications, and to the progression towards heart failure [63]. On the other hand, circulating levels of IL-18 are a predictor of CV events in subjects with coronary artery disease [64].

In recent years, another intriguing aspect that has been explored in gout pathogenesis is the relationship between inflammasome activation and the circadian rhythm [65]. It is well established that circadian rhythms affect the immune system, including the inflammasome pathways that, as mentioned above, are leading actors in gout pathogenesis. The notion that gout flares often occur late at night or during the early hours of the morning rather than during the day led investigators to explore how hormones whose levels are modulated by the circadian rhythms (namely by neurons of the ‘central clock’) such as melatonin, melanocortins and glucocorticoids could affect the inflammasome activation. Likewise, MSU crystals may modulate the gene expression of key molecules in macrophages and ultimately disrupt their ‘peripheral clock’. However, we are still far from a detailed understanding of these issues as highlighted by the differences observed in mouse models compared to humans and by the hurdles of in vitro studies (e.g., within a cell population isolated from a human being, there is a heterogenous mix of cells at different points in their circadian cycles).

**Figure 1:**
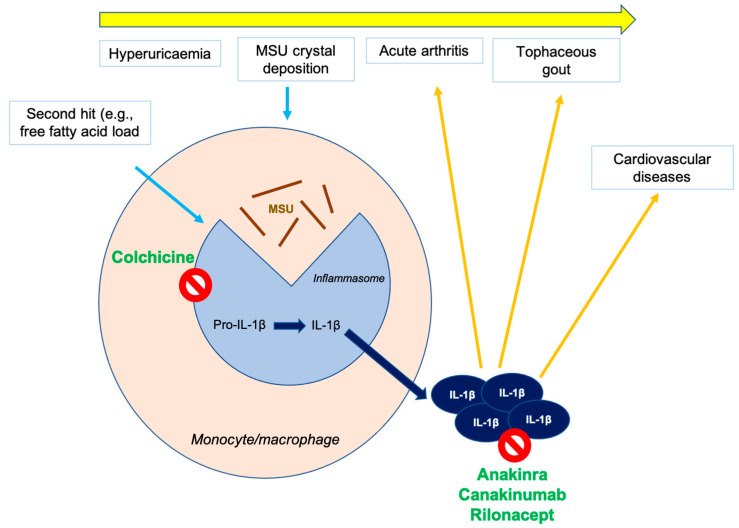
The pathogenesis of gout and the site of action of colchicine and targeted therapeutic agents. IL, interleukin; MSU, monosodium urate.

On this basis, and while we gather further knowledge on the inflammasome and its regulation, we acknowledge that the inhibition of the inflammasome and the downstream cytokines would allow us not only to act on the classical clinical features of gout but also on the CV risk of these patients. Over the years, the selective targeting of IL-1β has been pursued but current recommendations limit the use of these compounds for people with acute gout or who have frequent flares, or who have contraindications or have not responded adequately to the standard treatment of colchicine, non-steroidal anti-inflammatory drugs and glucocorticoids [66,67] (Figure 1). In this scenario, a leading actor may be colchicine, which has historically has been the cornerstone in the treatment of acute gouty arthritis and the prevention of recurrences. Colchicine, whose mechanism of action is to induce the depolymerisation of microtubules by binding to tubulin, is able to inhibit activation of the inflammasome and thus prevent the release of IL-1β and IL-18. In addition, since colchicine has a direct effect on mitosis (causing mitotic arrest) and mitosis exhibits circadian rhythm, the timing of colchicine administration could significantly influence its effects on cell division and tissue homeostasis. This complex interplay highlights the importance of considering not only the clinical repurposing of colchicine for CV prevention but also, and even more interestingly, the potential for time-of-day-dependent responses. This would allow the exploration of whether chronotherapeutic approaches (i.e., timing the drug administration) could optimize its benefits or minimize adverse effects [68].

From a CV perspective. Solomon et al., back in 2016, demonstrated that colchicine use was associated with a reduced risk of a CV events among patients with gout over a 16.5-month median follow-up [69]. More recently, a large study reported that among individuals with gout, those who experienced a CV event had significantly higher odds of a recent gout flare in the preceding days [70]. In this scenario, data on the CV effects of colchicine in the general population represented a milestone, opening potential new avenues in the holistic management of gout beyond articular manifestations.

The European Alliance of Associations for Rheumatology (EULAR) and the American College of Rheumatology (ACR), recommended flare prophylaxis, with colchicine as a first-line drug, during the first 3–6 months of urate-lowering therapy [66,67]. In this regard, a large retrospective study enrolling almost 100,000 patients diagnosed with gout between 1997 and 2021 and initiating urate-lowering therapy, demonstrated that the risk of CV events was reduced in those being prescribed colchicine prophylaxis compared with no prophylaxis [71]. These results are certainly encouraging, but as outlined by the authors themselves, should be interpreted with caution due to several limitations of the study. Nonetheless, since colchicine is a cheap and broad-spectrum compound with a powerful anti-inflammatory effect, its application as a therapeutic strategy for CV prevention in gout is of great interest. In fact, the need to target inflammation for a comprehensive management of CV risk was hampered by the availability of drugs such as IL-1 inhibitors, which was burdened by safety and economic issues [72]. Unfortunately, we are still far from a broad application of colchicine for CV prevention in clinical practice in gout. However, as we build real-life experience on the CV effects of colchicine while using it for gout flare prevention, we urge clinicians to share their experience with the scientific community and help to unmask the multifaceted potential of this drug in gout.

Finally, the potential benefit of colchicine for CV prevention in rheumatology practice goes well beyond gout, with the possibility to use it in addition to a background therapy that may already include anti-inflammatory or immunosuppressive drugs in patients with other chronic inflammatory diseases. However, the enthusiasm towards this approach has been dampened by the results of a recent systematic literature review identifying a huge knowledge gap. In fact, despite the wealth of data about colchicine for CV prevention in individuals with high CV risk, specific information about patients with conditions such as RA or PsA is lacking [73]. It is unclear if and how many patients with these diseases were recruited in clinical trials and therefore, we envisage and advocate for future studies selectively enrolling individuals with chronic inflammatory disease to shed some light on this matter.

## 9. Conclusions

Over the years, the scientific community has witnessed a continuous evolution of the CV prevention paradigm both in the general population and in specific patient subgroups such as those with chronic inflammatory diseases. It is now well established that UA is a pleiotropic molecule playing a key role in the pathogenesis of various conditions, including CV diseases. Therefore, it is conceivable to speculate that existing guidelines will soon be updated to incorporate novel cut-off values for pharmacological treatment from a CV perspective. In parallel we envisage that colchicine will move to the forefront of CV prevention and hopefully, as additional evidence accrues, be broadly implemented in clinical practice to improve the morbidity and mortality of CV diseases. This would allow us to raise the bar in CV prevention not only in the general population but also, and particularly, in individuals with chronic inflammatory diseases, since CV diseases are still a leading cause of death in these patients.

## Data Availability

No new data were created.

## Abbreviations

The following abbreviations are used in this manuscript:

ACR	American College of Rheumatology
CASPAR	Classification Criteria for Psoriatic Arthritis
CI	Confidence interval
CV	Cardiovascular
EULAR	European Alliance of Associations for Rheumatology
HR	Hazard ratio
IL	Interleukin
ILD	Interstitial lung disease
LoDoCo	Low-dose colchicine
MACE	Major cardiovascular events
MASLD	Metabolic dysfunction-associated steatotic liver disease
MSU	Monosodium urate
NAFLD	Non-alcoholic fatty liver disease
NHANES	National Health and Nutrition Examination Survey
OR	Odds ratio
PsA	Psoriatic arthritis
PsO	Psoriasis
RA	Rheumatoid arthritis
SD	Sjogren disease
SLE	Systemic lupus erythematosus
SSc	Systemic sclerosis
SUA	Serum uric acid
TNF	Tumour necrosis factor
UA	Uric acid
URRAH	Uric Acid Right for Heart Health

## References

[1] Du L., Zong Y., Li H., Wang Q., Xie L., Yang B., Pang Y., Zhang C., Zhong Z., Gao J. (2024). Hyperuricemia and its related diseases: Mechanisms and advances in therapy. Signal Transduct. Target. Ther..

[2] Li Q., Li X., Wang J., Liu H., Kwong J.S., Chen H., Li L., Chung S.C., Shah A., Chen Y. (2019). Diagnosis and treatment for hyperuricemia and gout: A systematic review of clinical practice guidelines and consensus statements. BMJ Open..

[3] Feig D.I., Kang D.H., Johnson R.J. (2008). Uric acid and cardiovascular risk. N. Engl. J. Med..

[4] Williams B., Mancia G., Spiering W., Agabiti Rosei E., Azizi M., Burnier M., Clement D.L., Coca A., de Simone G., Dominiczak A. (2018). ESC Scientific Document Group; 2018 ESC/ESH Guidelines for the management of arterial hypertension. Eur. Heart J..

[5] Ridker P.M., Cushman M., Stampfer M.J., Tracy R.P., Hennekens C.H. (1997). Inflammation, aspirin and the risk of cardiovascular disease in apparently healthy men. N. Engl. J. Med..

[6] Hansildaar R., Vedder D., Baniaamam M., Tausche A.-K., Gerritsen M., Nurmohamed M.T. (2021). Cardiovascular risk in inflammatory arthritis: Rheumatoid arthritis and gout. Lancet Rheumatol..

[7] Alunno A., Carubbi F., Rodríguez-Carrio J., Gossec L., Donohoe S., Ferri C. (2024). The management of cardiovascular risk in psoriatic disease: A bridge over troubled water. Semin. Arthritis Rheum..

[8] Chowalloor P., Raymond W.D., Cheah P., Keen H. (2020). The burden of subclinical intra-articular inflammation in gout. Int. J. Rheum. Dis..

[9] Saito Y., Tanaka A., Node K., Kobayashi Y. (2021). Uric acid and cardiovascular disease: A clinical review. J. Cardiol..

[10] Shubietah A., Awashra A., Milhem F., Ghannam M., Hattab M., Rajab I., Neiroukh H., Zahdeh M., Nouri A., Assaassa A. (2025). Hyperuricemia and Cardiovascular Risk: Insights and Implications. Crit. Pathw. Cardiol..

[11] Yuan H., Yu C., Li X., Zhu X., Zhao C., Zhang Z., Yang Z. (2015). Serum uric acid levels and risk of metabolic syndrome: A dose–response meta-analysis of prospective studies. J. Clin. Endocrinol. Metabolism.

[12] Virdis A., Masi S., Casiglia E., Tikhonoff V., Cicero A.F.G., Ungar A., Rivasi G., Salvetti M., Barbagallo C.M., Bombelli M. (2020). Working Group on Uric Acid and Cardiovascular Risk of the Italian Society of Hypertension; Identification of the Uric Acid Thresholds Predicting an Increased Total and Cardiovascular Mortality Over 20 Years. Hypertension.

[13] Muiesan M.L., Salvetti M., Virdis A., Masi S., Casiglia E., Tikhonoff V., Barbagallo C.M., Bombelli M., Cicero A.F.G., Cirillo M. (2021). Working Group on Uric Acid and Cardiovascular Risk of the Italian Society of Hypertension. Serum uric acid, predicts heart failure in a large Italian cohort: Search for a cut-off value the URic acid Right for heArt Health study. J. Hypertens..

[14] Ying H., Yuan H., Tang X., Guo W., Jiang R., Jiang C. (2021). Impact of Serum Uric Acid Lowering and Contemporary Uric Acid-Lowering Therapies on Cardiovascular Outcomes: A Systematic Review and Meta-Analysis. Front. Cardiovasc. Med..

[15] Mackenzie I.S., Hawkey C.J., Ford I., Greenlaw N., Pigazzani F., Rogers A., Struthers A.D., Begg A.G., Wei L., Avery A.J. (2022). Allopurinol versus usual care in UK patients with ischaemic heart disease (ALL-HEART): A multicentre, prospective, randomised, open-label, blinded-endpoint trial. Lancet.

[16] Ye Y., Liao G., Liu T., Hu X., Chen X., Bai L., Peng Y. (2023). Allopurinol for Secondary Prevention in Patients with Cardiovascular Disease: A Systematic Review and Meta-Analysis of Randomized Controlled Trials. J. Cardiovasc. Dev. Dis..

[17] Kijima S., Matsui K., Hiramitsu S., Hidatome I., Waki M., Uchiyama K., Yokota N., Tokutake E., Wakasa Y., Jinnouchi H. (2019). Febuxostat for Cerebral and CaRdiorenovascular Events PrEvEntion StuDy Randomized Controlled Trial. Eur. Heart J..

[18] Piani F., Agnoletti D., Borghi C. (2023). Advances in pharmacotherapies for hyperuricemia. Expert Opin. Pharmacother..

[19] Banerjee M., Pal R., Maisnam I., Chowdhury S., Mukhopadhyay S. (2023). Serum uric acid lowering and effects of sodium-glucose cotransporter-2 inhibitors on gout: A meta-analysis and meta-regression of randomized controlled trials. Diabetes Obes. Metab..

[20] You Y., Zhao Y., Chen M., Pan Y., Luo Z. (2023). Effects of empagliflozin on serum uric acid level of patients with type 2 diabetes mellitus: A systematic review and meta-analysis. Diabetol. Metab. Syndr..

[21] Zhang L., Zhang F., Bai Y., Huang L., Zhong Y., Zhang X. (2024). Effects of sodium-glucose cotransporter-2 (SGLT-2) inhibitors on serum uric acid levels in patients with chronic kidney disease: A systematic review and network meta-analysis. BMJ Open Diabetes Res. Care.

[22] Packer M. (2024). Hyperuricemia and Gout Reduction by SGLT2 Inhibitors in Diabetes and Heart Failure: JACC Review Topic of the Week. J. Am. Coll. Cardiol..

[23] Alfaddagh A., Martin S.S., Leucker T.M., Michos E.D., Blaha M.J., Lowenstein C.J., Jones S.R., Toth P.P. (2020). Inflammation and cardiovascular disease: From mechanisms to therapeutics. Am. J. Prev. Cardiol..

[24] Willerson J.T., Ridker P.M. (2004). Inflammation as a cardiovascular risk factor. Circulation.

[25] Ridker P.M., Everett B.M., Thuren T., MacFadyen J.G., Chang W.H., Ballantyne C., Fonseca F., Nicolau J., Koenig W., Anker S.D. (2017). CANTOS trial group. Antiinflammatory Therapy with Canakinumab for Atherosclerotic Disease. N. Engl. J. Med..

[26] Ridker P.M., Everett B.M., Pradhan A., MacFadyen J.G., Solomon D.H., Zaharris E., Mam V., Hasan A., Resenberg Y., Iturriaga E. (2019). CIRT investigators. Low-Dose Methotrexate for the Prevention of Atherosclerotic Events. N. Engl. J. Med..

[27] Perkovic V., Tuttle K.R., Sattar N., Lincoff A.M., Navar A.M., Marx N., Hvelplund A., Baeres F.M.M., Engelmann M.D., Hovingh G.K. (2025). Design of the ZEUS trial: Interleukin 6 inhibition with ziltivekimab for cardiovascular protection in chronic kidney disease. Kidney Int. Rep..

[28] Ridker P.M. (2021). From RESCUE to ZEUS: Will interleukin-6 inhibition with ziltivekimab prove effective for cardiovascular event reduction?. Cardiovasc. Res..

[29] Nidorf S.M., Eikelboom J.W., Budgeon C.A., Thompson P.L. (2013). Low-dose colchicine for secondary prevention of cardiovascular disease. J. Am. Coll. Cardiol..

[30] Nidorf S.M., Fiolet A.T.L., Mosterd A., Eikelboom J.W., Schut A., Opstal T.S.J., The S.H.K., Xy X.F., Ireland M.A., Lenderink T. (2020). LoDoCo2 trial investigators. Colchicine in Patients with Chronic Coronary Disease. N. Engl. J. Med..

[31] https://www.accessdata.fda.gov/drugsatfda_docs/label/2023/215727s000lbl.pdf.

[32] Visseren F.L.J., Mach F., Smulders Y.M., Carballo D., Koskinas K.C., Bäck M., Benetos A., Biffi A., Boavida J.M., Capodanno D. (2021). 2021 ESC Guidelines on cardiovascular disease prevention in clinical practice. Eur. Heart J..

[33] Dalbeth N., Gosling A.L., Gaffo A., Abishek A. (2021). Gout. Lancet.

[34] Dalbeth N., Choi H.K., Joosten L.A.B., Khanna P.P., Matsuo H., Perez-Ruiz F., Stamp L.S. (2019). Gout. Nat. Rev. Dis. Primers.

[35] van der Pol K.H., Wever K.E., Verbakel M., Visseren F.L.J., Cornel J.H., Rongen G.A. (2021). Allopurinol to reduce cardiovascular morbidity and mortality: A systematic review and meta-analysis. PLoS ONE.

[36] White W.B., Saag K.G., Becker M.A., Borer J.S., Gorelick P.B., Whelton A., Hunt B., Castillo M., Gunawardhana L. (2018). CARES Investigators Cardiovascular Safety of Febuxostat or Allopurinol in Patients with Gout. N. Engl. J. Med..

[37] Mackenzie I.S., Ford I., Nuki G., Hallas J., Hawkey C.J., Webster J., Ralston S.H., Walters M., Robertson M., De Caterina R. (2020). FAST study group. Long-term cardiovascular safety of febuxostat compared with allopurinol in patients with gout (FAST): A multicentre, prospective, randomised, open-label, non-inferiority trial. Lancet.

[38] Su C.Y., Shen L.J., Hsieh S.C., Lin L.Y., Lin F.J. (2019). Comparing Cardiovascular Safety of Febuxostat and Allopurinol in the Real World: A Population-Based Cohort Study. Mayo Clin. Proc..

[39] Zhang M., Solomon D.H., Desai R.J., Kang E.H., Liu J., Neogi T., Kim S.C. (2018). Assessment of Cardiovascular Risk in Older Patients with Gout Initiating Febuxostat Versus Allopurinol. Circulation.

[40] Zhao C., Xiao Q., Huang W., Chen Y., Yang X. (2025). Association between rheumatoid arthritis and hyperuricemia among adults: A cross-sectional study based on NHANES data. Clin. Rheumatol..

[41] Panoulas V.F., Douglas K.M., Milionis H.J., Nightingale P., Kita M.D., Klocke R., Metsios G.S., Stavropoulos-Kalinoglou A., Elisaf M.S., Kitas G.D. (2008). Serum uric acid is independently associated with hypertension in patients with rheumatoid arthritis. J. Hum. Hypertens..

[42] Panoulas V.F., Milionis H.J., Douglas K.M., Nightingale P., Kita M.D., Klocke R., Elisaf M.S., Kitas G.D. (2007). Association of serum uric acid with cardiovascular disease in rheumatoid arthritis. Rheumatology.

[43] Kuo D., Crowson C.S., Gabriel S.E., Matteson E.L. (2014). Hyperuricemia and Incident Cardiovascular Disease and Noncardiac Vascular Events in Patients with Rheumatoid Arthritis. Int. J. Rheumatol..

[44] Chiou A., England B.R., Sayles H., Thiele G.M., Duryee M.J., Baker J.F., Singh N., Cannon G.W., Kerr G.S., Reimold A. (2020). Coexistent Hyperuricemia and Gout in Rheumatoid Arthritis: Associations with Comorbidities, Disease Activity, and Mortality. Arthritis Care Res..

[45] Lyngdoh T., Marques-Vidal P., Paccaud F., Preisig M., Waeber G., Bochud M., Vollenweider P. (2011). Elevated serum uric acid is associated with high circulating inflammatory cytokines in the population-based Colaus study. PLoS ONE.

[46] Gasse P., Riteau N., Charron S., Girre S., Fick L., Pétrilli V., Tschopp J., Lagente V., Quesniaux V., Ryffel B. (2009). Uric acid is a danger signal activating NALP3 inflammasome in lung injury inflammation and fibrosis. Am. J. Respir. Crit. Care Med..

[47] Catrina A., Ytterberg J., Reynisdottr G., Malström V., Klareskog L. (2014). Lungs, joints and immunity against citrullinated proteins in rheumatoid arthritis. Nat. Rev. Rheumatol..

[48] Hu Z., Wang H., Huang J., Yang G., Luo W., Zhong J., Zheng X., Wei X., Luo X., Xiong A. (2024). Cardiovascular disease in connective tissue disease-associated interstitial lung disease: A systematic review and meta-analysis of observational studies. Autoimmun. Rev..

[49] FitzGerald O., Ogdie A., Chandran V., Coates L.C., Kavanaugh A., Tillett W., Leung Y.Y., deWit M., Scher J.U., Mease P.J. (2021). Psoriatic arthritis. Nat. Rev. Dis. Primer.

[50] Liu Z., Ma X., Chang T., Yao C., Song M., Biyue S., Zhang F., Liu J., Jiang Q. (2025). Associations between psoriasis, psoriatic arthritis and gout or hyperuricemia: A systematic review and meta-analysis. Am. J. Med. Sci..

[51] Yuan Y., Liu M., Liu W., Du H. (2019). The association of serum uric acid levels in psoriasis patients: A systematic review and network meta-analysis. Medicine (Baltimore).

[52] Petrie J.L., Patman G.L., Sinha I., Alexander T.D., Reeves H.L., Agius L. (2013). The rate of production of uric acid by hepatocytes is a sensitive index of compromised cell ATP homeostasis. Am. J. Physiol. Endocrinol. Metab..

[53] Sherri A., Mortada M.M., Makowska J., Sokolowska M., Lewandowska-Polak A. (2024). Understanding the interplay between psoriatic arthritis and gout: “Psout”. Rheumatol. Int..

[54] Gonzalez-Gay M.A., Gonzalez-Juanatey C., Vazquez-Rodriguez T.R., Gomez-Acebo I., Miranda-Filloy J.A., Paz-Carreira J., Martin J., Llorca J. (2009). Asymptomatic hyperuricemia and serum uric acid concentration correlate with subclinical atherosclerosis in psoriatic arthritis patients without clinically evident cardiovascular disease. Semin. Arthritis Rheum..

[55] AlJohani R., Polachek A., Ye J.Y., Chandran V., Gladman D.D. (2018). Characteristic and Outcome of Psoriatic Arthritis Patients with Hyperuricemia. J. Rheumatol..

[56] Wise E., Lewis E., Khanna P., Zhao L., McCune W.J. A Strong Association Between Gout and Diuretic Use Among Lupus Patients. Proceedings of the 2015 ACR/ARHP Annual Meeting.

[57] Yang Z., Liang Y., Xi W., Zhu Y., Li C., Zhong R. (2011). Association of serum uric acid with lupus nephritis in systemic lupus erythematosus. Rheumatol. Int..

[58] Ugolini-Lopes M.R., Gavinier S.S., Leon E., Viana V.T., Ferreira Borba E., Bonfà E. (2019). Is serum uric acid a predictor of long-term renal outcome in lupus nephritis?. Clin. Rheumatol..

[59] Elera-Fitzcarrald C., Reátegui-Sokolova C., Gamboa-Cardenas R.V., Medina M., Zevallos F., Pimentel-Quiroz V.R., Cucho-Venegas J.M., Alfaro-Lozano J., Rodriguez-Bellido Z., Pastor-Asurza C.A. (2020). Serum uric acid is associated with damage in patients with systemic lupus erythematosus. Lupus Sci. Med..

[60] Gigante A., Barbano B., Barilaro G., Quarta S., Gasperini M.L., Di Mario F., Romaniello A., Amoroso A., Cianci R., Rosato E. (2016). Serum uric acid as a marker of microvascular damage in systemic sclerosis patients. Microvasc. Res..

[61] Alunno A., Carubbi F., Mariani F.M., Martini C., Campanozzi E., Ferri C. (2023). The Interplay between Cardiovascular Risk, Cardiovascular Events, and Disease Activity in Primary Sjögren’s Syndrome: Is Uric Acid the Missing Link?. Nutrients.

[62] Feist E., Mitrovic S., Fautrel B. (2018). Mechanisms, biomarkers and targets for adult-onset Still’s disease. Nat. Rev. Rheumatol..

[63] Abbate A., Toldo S., Marchetti C., Kron J., Van Tassel B.W., Dinarello C.A. (2020). Interleukin-1 and the Inflammasome as Therapeutic Targets in Cardiovascular Disease. Circ. Res..

[64] Ridker P.M., MacFadyen J.G., Thuren T., Libby P. (2020). Residual inflammatory risk associated with interleukin-18 and interleukin-6 after successful interleukin-1beta inhibition with canakinumab: Further rationale for the development of targeted anti-cytokine therapies for the treatment of atherothrombosis. Eur. Heart J..

[65] Poulsen R.C., Dalbeth N. (2024). Circadian Rhythms in NLRP3 Inflammasome Regulation: Possible Implications for the Nighttime Risk of Gout Flares. Gout Urate Cryst. Depos. Dis..

[66] Richette P., Doherty M., Pascual E., Barskova V., Becce F., Castañeda-Sanabria J., Coyfish M., Guillo S., Jansen T.L., Janssens H. (2017). 2016 updated EULAR evidence-based recommendations for the management of gout. Ann. Rheum. Dis..

[67] FitzGerald J.D., Dalbeth N., Mikuls T., Brignardello-Petersen R., Guyatt G., Abeles A.M., Gelber A.C., Harrold L.R., Khanna D., King C. (2020). 2020 American College of Rheumatology guideline for the management of gout. Arthritis Care Res..

[68] Jacob H., Curtis A.M., Kearney C.J. (2020). Therapeutics on the clock: Circadian medicine in the treatment of chronic inflammatory diseases. Biochem. Pharmacol..

[69] Solomon D.H., Liu C.C., Kuo I-Hsin Zak A., Kim S.C. (2016). Effects of colchicine on risk of cardiovascular events and mortality among patients with gout: A cohort study using electronic medical records linked with Medicare claims. Ann. Rheum. Dis..

[70] Cipolletta E., Tata L.J., Nakafero G., Avery A.J., Mamas A.M., Abhishek A. (2022). Association Between Gout Flare and Subsequent Cardiovascular Events Among Patients with Gout. JAMA.

[71] Cipolletta E., Nakafero G., McCormick N., Yokose C., Avery A.J., Mamas A.M. (2025). Cardiovascular events in patients with gout initiating urate-lowering therapy with or without colchicine for flare prophylaxis: A retrospective new-user cohort study using linked primary care, hospitalisation, and mortality data. Lancet Rheumatol..

[72] Alunno A., Carubbi F., Ferri C. (2024). Colchicine and cardiovascular prevention. Eur. J. Intern. Med..

[73] Alunno A., Carubbi F., Martini C., Moronti V., Santilli J., Schoones J.W., Mariani F.M., Di Ruscio E., Altieri P., Ferri C. (2024). A systematic literature review of randomised controlled trials evaluating colchicine for cardiovascular prevention: There is an elephant in the room. Eur. J. Intern. Med..

